# Comparison of fistulotomy and fistulotomy in managing low anal fistula: A prospective study

**DOI:** 10.6026/973206300220348

**Published:** 2026-01-31

**Authors:** Upendra Singh, Kaushlendra Singh Narwariya, Krishna Pratap Singh, Pooja Rajput, Parul Nema, Vishnu Kumar Gupta

**Affiliations:** 1 Department of General Surgery, Government Medical College, Satna, Madhya Pradesh, India; 2 Department of General Surgery, S.R.V.S. Medical College, Shivpuri, Madhya Pradesh, India; 3 Department of Surgery, Motilal Nehru Medical College, Prayagraj, Uttar Pradesh, India; 4 Consultant Radiologist, Sai diagnostic Centre and Surgical care, Shivpuri, Madhya Pradesh, India; 5 Department of Pathology, SRVS Medical College, Shivpuri, Madhya Pradesh, India; 6 Department of Community Medicine, SRVS Medical College, Shivpuri, Madhya Pradesh, India

**Keywords:** Low anal fistula, fistulotomy, fistulectomy, wound healing, recurrence

## Abstract

Low anal fistula is a common anorectal disorder and fistulectomy and fistulotomy are frequently used surgical treatments. Therefore, it is of interest to evaluate the clinical outcomes, complications and recurrence rates between these two approaches in 120 patients with low anal fistula. Patients were randomly divided into two groups, with 60 undergoing fistulectomy and 60 undergoing fistulotomy. The study found that fistulotomy had significantly lower operative times, postoperative pain and faster wound healing compared to fistulectomy. There was no significant difference in recurrence or continence between the two groups. Fistulotomy proved to be a simpler, quicker and less painful procedure, making it the preferred option in selected cases.

## Background:

A fistula-in-ano is defined as an abnormal epithelialized tract that forms between the anal canal and the perianal skin, most commonly resulting from cryptoglandular infection and subsequent abscess formation. Low anal fistulas-those involving minimal external sphincter muscle and lying superficial to or traversing only the lower portion of the external sphincter-present a frequently encountered benign anorectal condition, yet optimal surgical management remains debated. Recent epidemiological data suggest that simple, low fistulas account for the majority of fistula-in-ano cases and that minimizing morbidity while maintaining high cure rates is the therapeutic priority [1]. Surgical options for low anal fistula include fistulotomy, in which the tract is laid open and allowed to heal by secondary intention and fistulectomy, in which the entire tract is excised. Proponents of fistulotomy highlight its simplicity, shorter operative time and faster healing, with low rates of incontinence in appropriately selected patients [2]. Conversely, advocates of fistulectomy argue that complete excision of the tract may reduce the risk of recurrence and residual sepsis, albeit at the cost of greater tissue disruption and longer healing times [3]. Although previous meta-analyses and randomized controlled trials have compared the two techniques, results remain inconclusive. One recent systematic review found no statistically significant difference in healing time or recurrence between the two methods, but identified greater operative trauma and longer healing associated with fistulectomy [4]. Other prospective series have reported faster wound healing and lower postoperative pain with fistulotomy, though they raise concerns about potential incontinence when the tract involves more sphincter muscle [5, 6]. Therefore, it is of interest to compare healing time, postoperative pain, wound complications, sphincter preservation and short-term recurrence between fistulectomy and fistulotomy in patients with low anal fistula.

## Materials and Methods:

## Study design and setting:

This prospective comparative study was conducted in at a tertiary care teaching hospital. The study aimed to evaluate and compare the outcomes of fistulectomy and fistulotomy in the management of low anal fistula. Informed written consent was taken from all participants.

## Study population:

A total of 120 patients diagnosed with low anal fistula were included in the study. Participants were randomly allocated into two equal groups of 60 each, based on a computer-generated randomization sequence. Patients in Group A underwent fistulectomy, whereas those in Group B were treated by fistulotomy. The diagnosis of low anal fistula was confirmed through clinical examination and, when indicated, by MRI fistulography to delineate the tract anatomy.

## Inclusion and exclusion criteria:

The study included adult patients aged between 18 and 65 years who had a single, uncomplicated, primary low anal fistula. Patients presenting with high anal or complex fistulas, recurrent fistulas, or associated systemic diseases such as Crohn's disease, tuberculosis, or diabetes mellitus were excluded. Individuals with a history of previous anal surgery, immunocompromised status, or that receiving long-term corticosteroid therapy were also not considered eligible. Pregnant women were excluded from participation to avoid potential surgical risks.

## Preoperative assessment:

All patients underwent a detailed history and thorough clinical examination, including digital rectal examination and proctoscopic evaluation to confirm the internal opening and course of the fistulous tract. Baseline laboratory investigations such as complete blood count, fasting blood sugar and coagulation profile were performed. The site of the external opening and its relation to the anal verge were carefully documented to guide the choice of operative technique.

## Surgical procedure:

All operations were performed under spinal or regional anesthesia by surgeons with experience in colorectal procedures. In the fistulectomy group, the entire fistulous tract was excised in to using a probe-guided dissection method, ensuring complete removal of the epithelialized tract and internal opening. Hemostasis was achieved meticulously and the wound was left open for secondary healing. In contrast, the fistulotomy group underwent laying open of the tract along the probe using electrocautery, followed by gentle curettage of granulation tissue. The wound edges were marsupialized to promote rapid epithelialization and minimize dead space formation.

## Postoperative management and follow-up:

Postoperative care was standardized for both groups. All patients received prophylactic antibiotics for five days, analgesics for pain control and sitz baths twice daily starting from the second postoperative day. The wounds were examined on the second postoperative day and subsequently during weekly follow-up visits until complete healing were achieved. Patients were followed up for a period of three months to assess wound healing, postoperative pain, infection, incontinence and recurrence. Pain intensity was assessed using the Visual Analogue Scale (VAS) on postoperative days 1, 3 and 7, while wound healing was defined as complete epithelialization of the surgical site. Anal continence was evaluated using the Wexner incontinence score during each follow-up visit.

## Outcome measures:

The primary outcomes of the study were the time required for complete wound healing and the recurrence rate within the three-month follow-up period. Secondary outcomes included the degree of postoperative pain, incidence of wound infection and the occurrence of anal incontinence. All parameters were recorded using a predesigned proforma to ensure consistency and reliability.

## Statistical analysis:

The collected data were entered into Microsoft Excel and analyzed using Statistical Package for the Social Sciences (SPSS) version 25.0 (IBM Corp., USA). Quantitative variables were expressed as mean ± standard deviation and compared using the Student's t-test, whereas categorical variables were analyzed using the Chi-square test or Fisher's exact test, as applicable. A p-value less than 0.05 were considered statistically significant.

## Results:

The demographic and baseline parameters of both groups were comparable at the time of enrolment, ensuring that observed outcome differences were attributable to the surgical intervention rather than patient characteristics. As shown in Table 1, there were no statistically significant variations between the two groups regarding mean age, sex distribution, body mass index, duration of symptoms, or type of fistula tract. This homogeneity supports the validity of subsequent comparisons between fistulectomy and fistulotomy outcomes. Operative details demonstrated notable procedural differences. According to Table 2, the mean operative time and intraoperative blood loss were significantly higher in the fistulectomy group compared with the fistulotomy group. Similarly, the duration of hospital stay was shorter following fistulotomy, reflecting it's relatively simpler technique and lesser tissue dissection. These findings suggest that fistulotomy offers an advantage in terms of operative efficiency and recovery time. Postoperative outcomes showed significant variation in pain perception and wound healing between the two procedures. As illustrated in Table 3, patients who underwent fistulotomy experienced lower pain scores during the first postoperative week. The healing period was also shorter in this group, indicating faster epithelialization and tissue repair. Despite these differences, the incidence of postoperative anal incontinence was comparable and minimal in both groups, suggesting that neither technique compromised sphincter integrity when performed correctly. Evaluation of postoperative complications revealed that wound infection and delayed healing were slightly more frequent after fistulectomy, although these differences were not statistically significant. As presented in Table 4, the overall rate of complications was low and both techniques demonstrated satisfactory safety profiles. The short-term recurrence rates at three months were minimal and comparable, reaffirming that both approaches achieve effective tract eradication when appropriately selected and executed.

## Discussion:

The present prospective study demonstrated that fistulotomy offers significant advantages over fistulectomy in terms of shorter operative duration, reduced postoperative pain and faster wound healing time, while maintaining comparable rates of incontinence and short-term recurrence. These findings align with recent meta-analyses and clinical series which suggest that for simple low anal fistulae, laying open the tract (fistulotomy) may present a more efficient and patient-friendly alternative without compromising the overall efficacy of fistula eradication [7, 8]. Our data indicate that patients undergoing fistulotomy required significantly less operative time and had faster return to wound closure compared with those undergoing excisional fistulectomy - a pattern mirrored in several recent randomized cohorts. For example, in a cohort of 50 patients randomized to fistulotomy vs fistulectomy, the fistulotomy group had a markedly shorter hospital stay and lower postoperative pain scores [9, 10]. The reduced surgical trauma inherent in fistulotomy-due to minimal dissection and less tissue excision-likely underlies the faster recovery and lower pain observed. While the primary aim of fistulectomy is complete excision of the fistulous tract (and theoretically a lower risk of residual disease), the larger wound created and attendant increased tissue disruption may prolong healing and increase postoperative morbidity. Several systematic reviews have concluded that there is no statistically significant difference in recurrence rates and incontinence between the two techniques for low-lying fistulas, though heterogeneity remains in study quality and definitions [11, 12]. Our results reinforce this equivalence in efficacy, as our recurrence rates and incontinence incidences were low and similar between groups at three-month follow-up. It is worth noting that despite the overall equivalence in long- term efficacy, the choice of technique may be guided by patient-specific factors. For example, patients who are surgery-averse, frail, or demand early return to daily activities may particularly benefit from the simpler, less invasive fistulotomy approach. On the other hand, in selected cases where tract anatomy is complex-despite being classified as "low" by usual criteria-or where previous drainage history suggests a higher risk of residual tract, some surgeons may opt for fistulectomy to ensure full excision. However, the additional operative burden and slower healing associated with fistulectomy should be factored into decision-making. Our study bears certain limitations. The follow-up period of three months limits assessment of longer-term recurrence and anal continence outcomes; a longer surveillance period would strengthen the evidence for durable efficacy. Moreover, despite randomization, subtle variations in surgical technique, wound care practices and surgeon experience may contribute to outcome variability. Finally, assessment of long-term functional outcomes such as patient-reported continence quality of life and sphincter ultrasound evaluation were not included.

## Conclusion:

Both fistulectomy and fistulotomy are effective and safe procedures for the management of low anal fistula, with low recurrence and minimal postoperative complications. However, fistulotomy offers significant advantages in terms of reduced operative time, lesser postoperative pain and faster wound healing while maintaining comparable rates of continence preservation and recurrence. Therefore, fistulotomy may be considered the preferred surgical approach for uncomplicated low anal fistulas, provided that the sphincter involvement remains minimal and patient selection is appropriate.

## Figures and Tables

**Table 1:** Demographic and baseline characteristics of the study population (n = 120)

Parameter	Fistulectomy (n=60)	Fistulotomy (n=60)	p-value
Mean age (years)	41.8 ± 9.7	42.6 ± 10.1	0.68
Male : Female ratio	52:08:00	50:10:00	0.61
Mean BMI (kg/m^2^)	23.5 ± 2.9	23.2 ± 3.1	0.72
Mean duration of symptoms (months)	7.6 ± 3.2	7.4 ± 2.8	0.79
Type of fistula (intersphincteric / transsphincteric)	36 / 24	34 / 26	0.7

**Table 2:** Operative parameters and hospital stay

Parameter	Fistulectomy (n=60)	Fistulotomy (n=60)	p-value
Mean operative time (minutes)	42.5 ± 8.4	34.7 ± 6.9	<0.001
Mean intraoperative blood loss (mL)	48.2 ± 15.6	38.9 ± 12.7	0.002
Mean hospital stay (days)	2.9 ± 1.0	2.1 ± 0.7	<0.001

**Table 3:** Postoperative pain, healing time and incontinence

Parameter	Fistulectomy (n=60)	Fistulotomy (n=60)	p-value
Mean VAS pain score (Day 1)	6.3 ± 1.1	5.1 ± 1.0	<0.001
Mean VAS pain score (Day 3)	4.8 ± 1.0	3.7 ± 0.9	<0.001
Mean VAS pain score (Day 7)	3.1 ± 0.8	2.6 ± 0.7	0.003
Mean time to complete healing (weeks)	7.2 ± 1.4	5.6 ± 1.2	<0.001
Anal incontinence (Wexner score > 0)	3 (5.0%)	2 (3.3%)	0.65

**Table 4:** Postoperative complications and recurrence

Complication	Fistulectomy (n=60)	Fistulotomy (n=60)	p-value
Wound infection	6 (10.0%)	4 (6.7%)	0.51
Secondary bleeding	2 (3.3%)	1 (1.7%)	0.56
Delayed wound healing (>8 weeks)	5 (8.3%)	2 (3.3%)	0.24
Recurrence at 3 months	2 (3.3%)	1 (1.7%)	0.56
